# Effects of low-stearate palm oil and high-stearate lard high-fat diets on rat liver lipid metabolism and glucose tolerance

**DOI:** 10.1186/s12986-015-0053-y

**Published:** 2015-12-18

**Authors:** Sharon Janssens, Mattijs M. Heemskerk, Sjoerd A. van den Berg, Natal A. van Riel, Klaas Nicolay, Ko Willems van Dijk, Jeanine J. Prompers

**Affiliations:** Biomedical NMR, Department of Biomedical Engineering, Eindhoven University of Technology, PO Box 513, 5600 MB Eindhoven, The Netherlands; Department of Human Genetics, Leiden University Medical Center, Leiden, The Netherlands; Computational Biology, Department of Biomedical Engineering, Eindhoven University of Technology, Eindhoven, The Netherlands; Department of Medicine, division Endocrinology, Leiden University Medical Center, Leiden, The Netherlands; Present address: Amphia Hospital, Breda, The Netherlands

**Keywords:** Diet and dietary lipids, Fatty acid metabolism, Fatty acid oxidation, Insulin resistance, Magnetic resonance spectroscopy, Mitochondria, Nutrition, Obesity, Triglycerides, VLDL

## Abstract

**Background:**

Excess consumption of energy-dense, high-fat Western diets contributes to the development of obesity and obesity-related disorders, such as fatty liver disease. However, not only the quantity but also the composition of dietary fat may play a role in the development of liver steatosis. The aim of this study was to determine the effects of low-stearate palm oil and high-stearate lard high-fat diets on in vivo liver lipid metabolism.

**Methods:**

Wistar rats were fed with either normal chow (CON), a high-fat diet based on palm oil (HFP), or a high-fat diet based on lard (HFL). After 10 weeks of diet, magnetic resonance spectroscopy was applied for the in vivo determination of intrahepatocellular lipid content and the uptake and turnover of dietary fat after oral administration of ^13^C-labeled lipids. Derangements in liver lipid metabolism were further assessed by measuring hepatic very-low density lipoprotein (VLDL) secretion and *ex vivo* respiratory capacity of liver mitochondria using fat-derived substrates. In addition, whole-body and hepatic glucose tolerance were determined with an intraperitoneal glucose tolerance test.

**Results:**

Both high-fat diets induced liver lipid accumulation (*p* < 0.001), which was accompanied by a delayed uptake and/or slower turnover of dietary fat in the liver (*p* < 0.01), but without any change in VLDL secretion rates. Surprisingly, liver lipid content was higher in HFP than in HFL (*p* < 0.05), despite the increased fatty acid oxidative capacity in isolated liver mitochondria of HFP animals (*p* < 0.05). In contrast, while both high-fat diets induced whole-body glucose intolerance, only HFL impaired hepatic glucose tolerance.

**Conclusion:**

High-fat diets based on palm oil and lard similarly impair the handling of dietary lipids in the liver, but only the high-fat lard diet induces hepatic glucose intolerance.

## Background

A sedentary lifestyle and excess consumption of energy-dense Western diets contribute to the development of obesity and obesity-related disorders, such as insulin resistance and type 2 diabetes, dyslipidaemia, hypertension, cardiovascular disease, and fatty liver disease [1–3]. In obesity, the excessive amount of triglycerides (TG) is stored not only in adipose tissue, but also in muscle, liver, pancreas, and heart, which is referred to as ectopic lipid deposition [4, 5]. Hepatic steatosis is a hallmark of non-alcoholic fatty liver disease (NAFLD), the most common liver disorder in the Western world, which can evolve into non-alcoholic steatohepatitis (NASH), liver cirrhosis, and hepatocellular carcinoma [6–8]. The main pathways in hepatic lipid metabolism are uptake of fatty acids derived from dietary fat and adipose tissue TG, *de novo* lipogenesis, oxidation of fatty acids, and secretion of very-low density lipoproteins (VLDL). Hepatic steatosis occurs when the rate of uptake and synthesis of fatty acids by hepatocytes exceeds the rate of oxidation and secretion [7, 9, 10].

The metabolic fate of fatty acids depends on chain length, degree of saturation, and the stereoisomeric configuration of double bonds [11–15]. Therefore, dietary fats with different fatty acid compositions may differentially affect hepatic lipid metabolism. Data from the National Health and Nutrition Examination Survey (NHANES) show that from the fat consumed by the US population about one-third is saturated fat [16], with palmitic acid (C16:0) and stearic acid (C18:0) accounting for the majority of saturated fatty acid intake [17]. The efficiency of oxidation of fatty acids decreases with increasing chain length and it has been shown that stearic acid is poorly oxidized by hepatocytes [18–21]. Moreover, it has been reported that stearate is a poor substrate for the esterification into TG and subsequent VLDL synthesis and secretion [22, 23]. Dietary fatty acids have also been shown to affect insulin sensitivity in a chain-length dependent manner [24]. Mice receiving a high-fat diet based on lard developed both hepatic and peripheral insulin resistance, whereas mice fed with a high-fat diet based on palm oil developed peripheral insulin resistance only [25]. The two high-fat diets differed largely in the content of stearate, with the lard diet containing about 3-fold more stearate than the palm oil diet. Moreover, it was demonstrated that when the palm oil diet was supplemented with tristearin (to the stearate level of the lard based diet) hepatic insulin sensitivity was similarly impaired as with the lard diet, showing that this effect can be attributed to stearate per se [25]. Because of the low efficiency for oxidation and secretion, diets rich in stearate may more rapidly induce hepatic steatosis as compared with palmitate, which could contribute to hepatic insulin resistance through lipid-induced mechanisms [26]. However, the exact effects of stearate versus palmitate on hepatic lipid metabolism are currently not known.

Proton magnetic resonance spectroscopy (^1^H MRS) has proven to be a valuable tool for the non-invasive quantification of intrahepatocellular lipids (IHCL) [27–29]. Although it is a very powerful method to detect hepatic steatosis, it cannot discriminate between disturbances in lipid accretion on one hand and lipid utilization on the other. For this purpose, we previously introduced the application of proton-observed, carbon-edited (^1^H-[^13^C]) MRS in combination with the oral administration of ^13^C-labeled lipids [30, 31]. Using this method it is possible to determine in vivo and at multiple time points the amount of diet-derived ^13^C-labeled lipids in the liver, which gives information on the time course of uptake and utilization of dietary lipids.

The aim of this study was to determine the effects of low-stearate palm oil and high-stearate lard high-fat diets on liver lipid metabolism and glucose tolerance in rats. Wistar rats were fed a high-fat diet based on palm oil or a high-fat diet based on lard for a period of 10 weeks. ^1^H-[^13^C] MRS was used for the in vivodetermination of total IHCL content and the ^13^C enrichment of IHCL at baseline and 4 h and 24 h after the oral administration of ^13^C-labeled lipids. Derangements in liver lipid utilization were further assessed by measuring hepatic VLDL-TG secretion rates and *ex vivo* respiratory capacity of liver mitochondria using fat-derived substrates. In addition, we determined whole-body and hepatic glucose tolerance.

## Methods

### Animals and diets

Adult male Wistar rats (348.2 ± 1.5 g, 11 weeks of age, n = 60; Charles River Laboratories, The Netherlands) were housed in pairs at 20 °C and 50 % humidity on a 12 h light-dark cycle with ad libitum access to food and water. After one week of acclimatization the rats were equally divided into three groups: a control group receiving normal chow (CON; 9 energy percent (En%) from fat, 67 En% from carbohydrates, 24 En% from protein; R/M-H diet, Ssniff Spezialdiäten GmbH, Soest, Germany), a second group receiving a high-fat palm oil diet (HFP; 45 En% from fat (39.4 En% from palm oil and 5.5 En% from soybean oil), 35 En% from carbohydrate, 20 En% from protein; Research Diet Services, Wijk bij Duurstede, The Netherlands), and a third group receiving a high-fat lard diet (HFL; 45 En% from fat (39.4 En% from lard and 5.5 En% from soybean oil), 35 En% from carbohydrate, 20 En% from protein; Research Diet Services, Wijk bij Duurstede, The Netherlands). In a previous study, the fatty acid composition of the total fat content of the high-fat diets was determined and details can be found in Table 2 of reference [25]. Based on these data, the high-fat palm oil diet contained 16 En% of palmitate (C16:0) and 2 En% of stearate (C18:0), while the high-fat lard diet contained 13 En% of palmitate (C16:0) and 7 En% of stearate (C18:0). Otherwise, fatty acid composition of the two high-fat diets was similar, except for linoleic acid (C18:2w6), which was more abundant in the high-fat palm oil diet as compared with the high-fat lard diet (7 En% versus 4 En%). It should be noted though that the high-fat diets used in the current study were from a different batch and therefore the fatty acid composition may slightly differ. Body weight and food intake were determined weekly. All rats received the diet for a period of 10 weeks, after which each dietary group was divided into two subgroups: one group (n = 9 per diet group) for MRS measurements and oxygen consumption measurements of isolated liver mitochondria, and one group (n = 11 per diet group) for intraperitoneal glucose tolerance tests (ipGTT) with deuterated glucose and determination of hepatic VLDL-TG secretion rates. All animal experiments were reviewed and approved by the Animal Ethics Committee of Maastricht University, The Netherlands.

### MRS experiments

To determine total (^12^C + ^13^C) IHCL content and natural abundance ^13^C enrichment of IHCL in liver after 10 weeks of diet, animals (n = 9 per diet group) were subjected to baseline ^1^H-[^13^C] MRS measurements. Two days later, rats received 1.5 g [U-^13^C] labeled algal lipid mixture (^13^C enrichment > 98 %; fatty acid composition: 53 % palmitic acid, 9 % palmitoleic acid, 28 % oleic acid, and 6 % linoleic acid; Buchem B.V., Apeldoorn, The Netherlands) per kg body weight orally. The following 4 h the rats remained fasted, after which ^1^H-[^13^C] MRS experiments were performed to determine ^13^C-enriched IHCL concentrations. Between 4 and 24 h after ^13^C-labeled lipid administration, the high-fat diet groups were pairwise fed with CON rats and therefore all rats consumed the same amount of calories. At 24 h, the final ^1^H-[^13^C] MRS experiments were performed. Blood samples were taken from the *vena saphena* after each MRS experiment and were collected in paraoxon-coated capillaries, to prevent lipolysis [32]. The samples were centrifuged at 1000 g for 10 min and plasma was frozen in liquid nitrogen and stored at -80 °C for the analysis of non-esterified fatty acids (NEFA) using the NEFA-HR(2) kit (Wako Chemicals, Neuss, Germany). After the last MRS measurement at 24 h, animals were euthanized by incising the *vena cava inferior*, and the median liver lobe was excised and used for the isolation of liver mitochondria.

During the MRS experiments, animals were anaesthetized using 1.5–2.5 % isoflurane (IsoFlo®; Abbott Laboratories Ltd, Maidenhead, Berkshire, UK). Body temperature was maintained at 37 ± 1 °C using heating pads. Respiration was monitored with a respiratory balloon and the respiration period was kept between 700 and 1200 ms. All MRS experiments were executed on a 6.3 T horizontal Bruker MR system (Bruker, Ettlingen, Germany). Localized ^1^H-[^13^C] MRS was performed on a 4x2x4 mm^3^ voxel in the median lobe of the liver using the LASER-POCE method as described previously [30]. Briefly, 3D localization of a voxel in the liver was achieved by the LASER (localization by adiabatic selective refocusing) sequence [33], which was combined with a POCE (proton-observed, carbon-edited) element [34, 35] for ^13^C editing. The ^13^C-editing pulse was centered on the lipid methylene resonance, as determined from an unlocalized ^13^C spectrum. For each voxel, 64 LASER-POCE experiments, consisting of 16 averages each, were performed serially in an interleaved fashion with the ^13^C-editing pulse turned on every other experiment. Water suppression was achieved using SWAMP (sequence for water suppression with adiabatic modulated pulses) [36]. As an internal reference, an unsuppressed water spectrum, consisting of 16 averages, was recorded from the same voxel. Other LASER-POCE parameters were as follows: TR = 2000 ms, TE = 26.8 ms, TE_POCE_ = 7.9 ms, number of data points = 640, WALTZ-16 ^13^C decoupling, and total scan time = 34 min.

Spectra from the 32 LASER-POCE experiments with and without the ^13^C-editing pulse were added separately, and the difference spectrum was calculated using Matlab (R2010a, Mathworks, Natick, MA, USA). Water and IHCL methylene (at 1.3 ppm) peak areas were quantified from the unsuppressed and suppressed spectra, respectively, using a nonlinear least-squares algorithm (AMARES) in the jMRUI software package. Total (^12^C + ^13^C) and ^13^C-labeled IHCL levels were calculated from the LASER-POCE spectra without ^13^C editing and the difference spectra, respectively, and are expressed as a percentage of the unsuppressed water signal measured in the same voxel. The average relative ^13^C enrichment determined at baseline was used to correct the absolute ^13^C-labeled IHCL levels at 4 and 24 h after ^13^C-labeled lipid administration for natural abundance of ^13^C, yielding the amount of lipids stored in the liver originating from the administered ^13^C-labeled lipids.

### Isolation of liver mitochondria

Liver mitochondria were isolated using a standard differential centrifugation procedure as described elsewhere [37]. Briefly, 1.13 ± 0.01 g of the excised median liver lobe (n = 27) was washed in ice cold 0.9 % KCl, removed from connective and adipose tissue, minced with scissors in medium A (250 mM sucrose, 10 mM Tris, 3 mM EGTA and 2 mg/ml BSA, pH 7.7 at 4 °C) and homogenized using a Potter-Elvehjem homogenizer. The homogenate was centrifuged at 800 g for 10 min at 4 °C. The supernatant was centrifuged at 7200 g for 10 min at 4 °C and the pellet was then resuspended in ice cold medium B (250 mM sucrose and 5 mM Tris, pH 7.3 at 4 °C) and centrifuged at 7200 g for 10 min at 4 °C. The mitochondrial pellet was resuspended in 250 μl of medium B. Protein content was determined using the BCA protein assay kit (Pierce, Thermo Fisher Scientific Inc., Rockford, IL, USA).

### Mitochondrial oxygen consumption rates

Oxygen consumption rates in isolated liver mitochondria were measured at 37 °C using a two-channel high-resolution Oroboros oxygraph-2 k (Oroboros, Innbruck, Austria). Mitochondria were incubated in assay medium containing 0.5 mM EGTA, 3 mM MgCl.6H_2_O, 60 mM K-lactobionate, 20 mM taurine, 10 mM KH_2_PO_4_, 20 mM HEPES, 110 mM sucrose, and 1 g∙l^-1^ BSA essentially fatty acid free, pH 7.1 at 30 °C. All measurements were performed in 2 ml of assay medium containing 20 μl of solution containing mitochondria. The following oxidizable substrates were used: (i) 5 mM pyruvate plus 5 mM malate (TCA cycle substrate), (ii) 25 μM palmitoyl-L-carnitine plus 2.5 mM malate (β-oxidation substrate independent of carnitine palmitoyltransferase 1 (CPT1)), and (iii) 25 μM palmitoyl-CoA plus 2.5 mM L-carnitine plus 2.5 mM malate (β-oxidation substrate dependent on CPT1). State 3 respiration, i.e. maximal ADP-stimulated oxygen consumption rate, was determined after adding 4.8 U∙ml^-1^ hexokinase, 12.5 mM glucose, and 1 mM ATP. State 4 respiration, i.e. basal oxygen consumption rate, was determined after the addition of 1.25 μM carboxyatractyloside (CAT) to fully block ATP synthesis. Respiratory control ratio (RCR) was calculated by dividing oxygen consumption rate in state 3 by oxygen consumption rate in state 4. Data acquisition and analysis was performed using DatLab software version 4.3 (Oroboros, Innsbruck, Austria).

### ipGTT with deuterated glucose

An intraperitoneal glucose tolerance test (ipGTT) was performed after 10 weeks of diet (n = 11 per diet group). After an overnight fast, rats received an intraperitoneal injection of glucose (1 mg/g body weight containing 50 % [6,6-^2^H_2_] glucose and 50 % glucose; in a 50 mg/ml concentration). Blood samples were taken from the *vena saphena* just before and at 7.5, 15, 30, 45, 60, 90 and 120 min after the injection. [6,6-^2^H_2_] glucose was > 98 % enriched (Buchem B.V., Apeldoorn, The Netherlands). Blood glucose concentration was determined using a glucose analyzer (Hemocue Diagnostics B.V., Waalre, The Netherlands).

Blood samples for analysis of insulin concentration were centrifuged at 1000 g for 10 min. The plasma aliquots were then frozen in liquid nitrogen and stored at -80 °C until analysis, which was performed using the Ultra Sensitive Rat Insulin ELISA kit (Crystal Chem, Inc. Downers Grove, IL, USA). The homeostatic model assessment for insulin resistance (HOMA-IR) index was calculated by multiplying fasting glucose and fasting insulin divided by 22.5 [38].

For the determination of plasma [6,6-^2^H_2_] glucose enrichment, blood was spotted onto filter paper (Sample Carrier Paper FT-2-460-460570, Sartorius, Goettingen, Germany). The dried blood spot was punched out from the middle area of the blood spot directly into a vial. Plasma glucose enrichments were determined as described previously [39, 40]. In short, plasma was deproteinized with 1 ml of methanol and evaporated after which the extract was derivatized to the glucose aldonitrile pentaacetate form. A sample of 1 μl was injected into the gas chromatograph/mass spectrometer (HP 6890 II GC System and 5973 Mass Selective Detector, Agilent Technologies, Palo Alto, CA, USA). Separation was achieved on a J&W DB17 capillary column (30 m × 0.25 mm × 0.25 μm; J&W Scientific, Folsom, CA, USA). Mass-over-charge ratios of 187, 188, and 189 were monitored in selective ion monitoring mode, from which the percentage of unlabeled and [6,6-^2^H_2_] glucose was calculated based on the theoretical isotopic distribution via multiple linear regression.

Areas under the ipGTT curves for plasma glucose (AUC_g_), insulin (AUC_i_), deuterated glucose (AUC_d2g_), and glucose enrichment (AUC_EN_) were calculated. In order to analyze the glucose enrichment (EN, % ^2^H_2_-glucose of total glucose) in more detail, a two equally sized-compartment model was used, roughly representing the intraperitoneal and the blood compartments, in Matlab. The model was then fitted to the glucose enrichment data at the different time points to estimate the peak height and peak time of plasma glucose enrichment.

### Hepatic VLDL-TG secretion rate

Two days after the ipGTT, rats were fasted for 4 h after which they were anaesthetized and given an intravenous injection of 400 mg/kg bodyweight Triton WR1339 (10 % (w/w) solution in phosphate buffered saline (PBS); Sigma-Aldrich, Zwijndrecht, The Netherlands) in the tail vein to inhibit lipoprotein lipase activity (and thereby blocking lipolysis). Animals remained anaesthetized during the whole experiment using 1.5-2.5 % isoflurane (IsoFlo®; Abbott Laboratories Ltd, Maidenhead, Berkshire, UK). Blood samples were taken from the *vena saphena* before and 30, 60, 90, 120 and 150 min after the injection of Triton WR1339 and plasma TG concentrations were determined at the different time points using the serum TG determination kit (Sigma-Aldrich, Zwijndrecht, The Netherlands). Hepatic VLDL-TG secretion rate was determined from the slope of a linear fit through the plasma TG versus time data and was corrected for body weight [41].

### Statistical analysis

Data are expressed as means ± standard error of the mean (SEM). Time course data of body weight, energy intake, ^13^C enrichment of IHCL, and plasma glucose, ^2^H_2_-glucose, glucose enrichment and insulin during the ipGTT were analyzed using mixed-model repeated-measures analysis of variance (ANOVA) with time point as the within-subjects factor and diet type (CON, HFP, and HFL) as the between-subjects factor, with Bonferroni corrected post-hoc tests. In case of a significant interaction term between time and diet, the effect of time was analyzed using a paired *t*-test (only for ^13^C enrichment of IHCL; for the other data time effects were not further analyzed) and differences between diets were evaluated by one-way ANOVA with Tukey HSD post-hoc analysis. The other data were analyzed using one-way ANOVA with Tukey HSD post-hoc analyses. Statistical analyses were performed using the IBM SPSS statistics 21 software package (SPSS Inc.; Chicago, IL, USA). Level of significance was set at *p* < 0.05.

## Results

### Animal characteristics

**Fig. 1:**
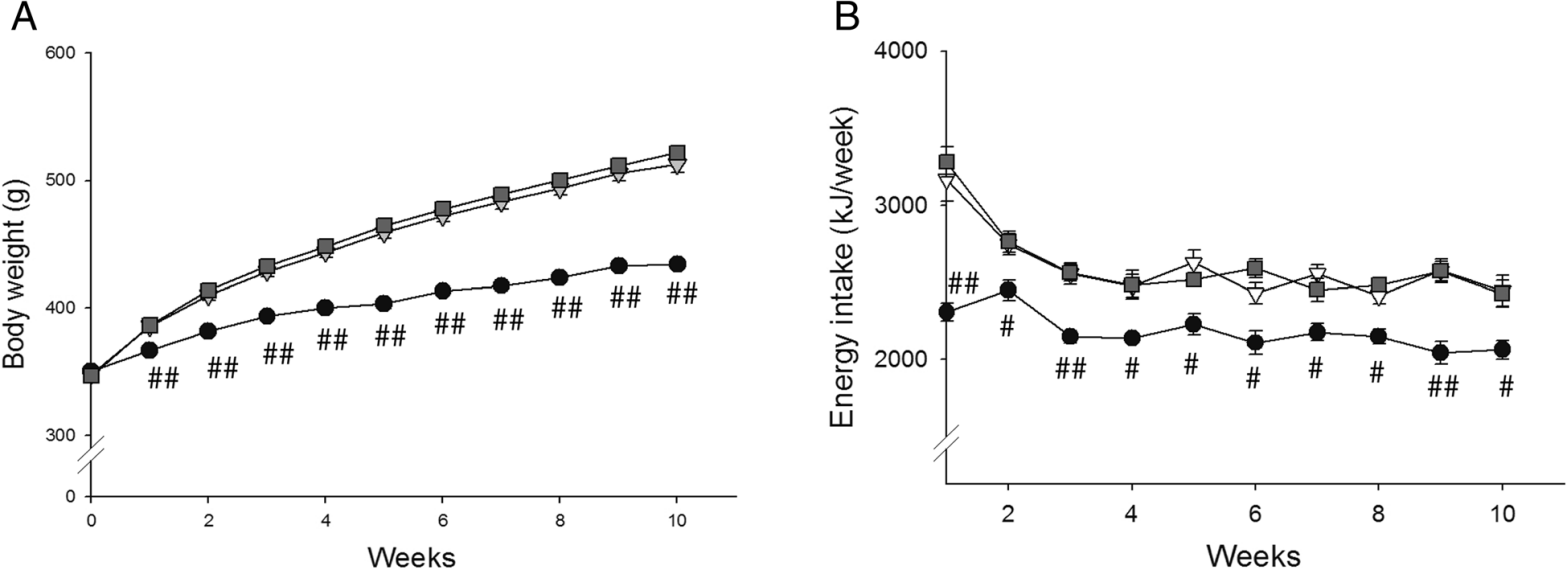
Body weight and energy intake during diet period. Body weight (**a**) and energy intake (**b**) of rats on normal chow control diet (●), high-fat palm oil diet (∇), and high-fat lard diet () determined weekly (n = 20 per group). Data are expressed as mean ± SEM. # *p* < 0.01, ## *p* < 0.001 vs. HFP and HFL

Figure 1 shows body weight and energy intake of the rats during the time course of the diets and Table 1 summarizes the animal characteristics over the 10-week diet period. Before the start of the diets, there was no difference in body weight between groups (Table 1). One week after starting the diets body weight was already higher in both high-fat diet groups and body weight remained higher in the high-fat diet groups compared with CON throughout the entire diet period, which was accompanied by a higher energy intake (Fig. 1; Table 1). However, there was no difference in body weight gain or energy intake between the two high-fat diet groups. After 10 weeks of diet, animals from the two high-fat diet groups had higher liver weights than CON animals (HFP vs. CON: p = 0.005; HFL vs. CON: p = 0.013), but liver weight did not differ between HFP and HFL animals. Plasma NEFA concentrations were not different between the three diet groups.

**Table 1:** Animal characteristics

	CON (n = 20)	HFP (n = 20)	HFL (n = 20)
Bodyweight before diet (g)	350 ± 2	348 ± 3	347 ± 3
Body weight after diet (g)	434 ± 3	513 ± 6 ***	522 ± 4 ***
Body weight gain (g)	84 ± 3	165 ± 6 ***	175 ± 4 ***
Energy intake (kJ/week)	2181 ± 15	2601 ± 45 ***	2613 ± 23 ***
Liver weight (g)	11.9 ± 0.2	12.9 ± 0.2 **	12.8 ± 0.2 *
Plasma NEFA (mM)	0.53 ± 0.02	0.53 ± 0.03	0.59 ± 0.03

Data are expressed as mean ± SEM. CON, normal chow control diet; HFP, high-fat palm oil diet; HFL, high-fat lard diet; NEFA, non-esterified fatty acid measured after 10 weeks of diet (n = 9 per diet group). * *p* < 0.05, ** *p* < 0.01, *** *p* < 0.001 vs. CON

### Magnetic resonance spectroscopy

**Fig. 2:**
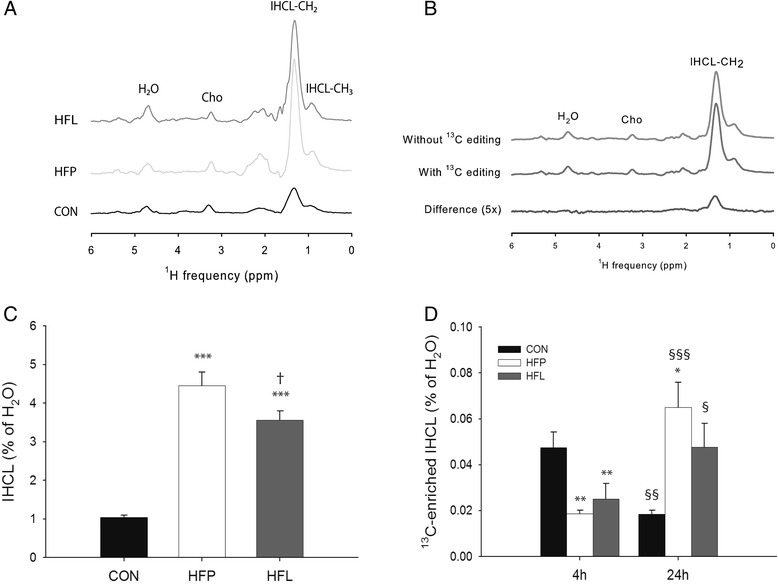
Determination of total IHCL content and ^13^C enrichment of IHCL by MRS. Typical examples of unedited LASER-POCE spectra acquired from the liver of animals from each diet group after 10 weeks of diet feeding (**a**), and LASER-POCE spectra with and without ^13^C editing acquired from the liver of a HFL rat at 24 h after ^13^C-labeled lipid administration, together with the calculated difference spectrum containing only ^13^C-coupled ^1^H resonances (5x magnification) (**b**). Total lipid content at baseline (**c**) and absolute ^13^C-labeled IHCL content corrected for ^13^C natural abundance at 4 and 24 h after the oral administration of [U-^13^C] algal lipid mixture (**d**) in rats on normal chow control diet (CON), high-fat palm oil diet (HFP), and high-fat lard diet (HFL) (n = 9 per group). H_2_O, residual water; Cho, choline; IHCL, intrahepatocellular lipids. Data are expressed as mean ± SEM. * *p* < 0.05, ** *p* < 0.01, *** *p* < 0.001 vs. CON; † *p* < 0.05 vs. HFP; § *p* < 0.05, §§ *p* < 0.01, §§ *p* < 0.001 vs. 4 h

Figure 2a displays typical examples of unedited ^1^H MR spectra acquired from the liver of animals from each diet group after 10 weeks of diet feeding, from which total IHCL content was determined. Both high-fat diets resulted in an elevated IHCL content compared with CON (*p* < 0.001; Fig. 2c). Moreover and surprisingly, IHCL content in HFP animals was higher than in HFL animals (*p* < 0.05).

Figure 2b shows an example of ^1^H MR spectra with and without ^13^C editing acquired from the liver of a HFL rat at 24 h after ^13^C-labeled lipid administration, and the calculated difference spectrum, from which the ^13^C enrichment of IHCL was determined. The relative ^13^C enrichment of IHCL before the administration of ^13^C-labeled lipids was not different between groups and was 1.23 ± 0.07 % on average (not shown). After correction for this natural abundance ^13^C enrichment, the absolute ^13^C-labeled IHCL content determined at 4 h after the administration of the ^13^C-labeled lipids was significantly lower in both high-fat diet groups compared with CON (*p* < 0.01; Fig. 2d). In CON, ^13^C-labeled IHCL content decreased 2.5-fold between 4 and 24 h after ^13^C-labeled lipid administration (*p* < 0.01). In contrast, between 4 and 24 h ^13^C-labeled IHCL content increased 3.5-fold in HFP (*p* < 0.001) and 1.9-fold in HFL (*p* < 0.05).

### Mitochondrial function

Oxygen consumption rates in isolated liver mitochondria oxidizing the glucose-derived substrate pyruvate plus malate were not different between diet groups (Table 2). In contrast, with the fat-derived substrates palmitoyl-L-carnitine plus malate and palmitoyl-CoA plus L-carnitine plus malate, state 3 oxygen consumption rates were higher in HFP comparted with CON (*p* < 0.05), while state 4 respiration was not different. Mitochondrial respiration with the fat-derived substrates was not significantly different in HFL compared with CON.

**Table 2:** Oxygen consumption rates in isolated liver mitochondria oxidizing different substrates in different metabolic states

	CON (n = 9)	HFP (n = 9)	HFL (n = 9)
Pyruvate + malate (nmol O_2_ ∙min^-1^∙mg protein^-1^)			
State 3	112 ± 7	143 ± 17	110 ± 11
State 4	18 ± 1	33 ± 7	19 ± 2
RCR	5.1 ± 0.5	5.1 ± 0.5	5.8 ± 0.7
Palmitoyl-L-carnitine + malate (nmol O_2_∙ min^-1^∙mg protein^-1^)			
State 3	162 ± 18	232 ± 22 *	193 ± 10
State 4	27 ± 3	30 ± 3	27 ± 3
RCR	6.0 ± 0.3	7.8 ± 1.2	7.9 ± 1.4
Palmitoyl-CoA + L-carnitine + malate (nmol O_2_ ∙min^-1^∙mg protein^-1^)			
State 3	134 ± 20	205 ± 18 *	151 ± 15
State 4	27 ± 5	26 ± 4	31 ± 7
RCR	4.6 ± 0.5	7.0 ± 0.8 *	4.7 ± 0.4 †

Data are expressed as mean ± SEM. CON, normal chow control diet; HFP, high-fat palm oil diet; HFL, high-fat lard diet; state 3, maximal ADP-stimulated oxygen consumption; state 4, basal oxygen consumption; RCR, respiratory control ratio. * *p* < 0.05 vs. CON; † *p* < 0.05 vs. HFP

### Hepatic VLDL triglyceride secretion rate

**Fig. 3:**
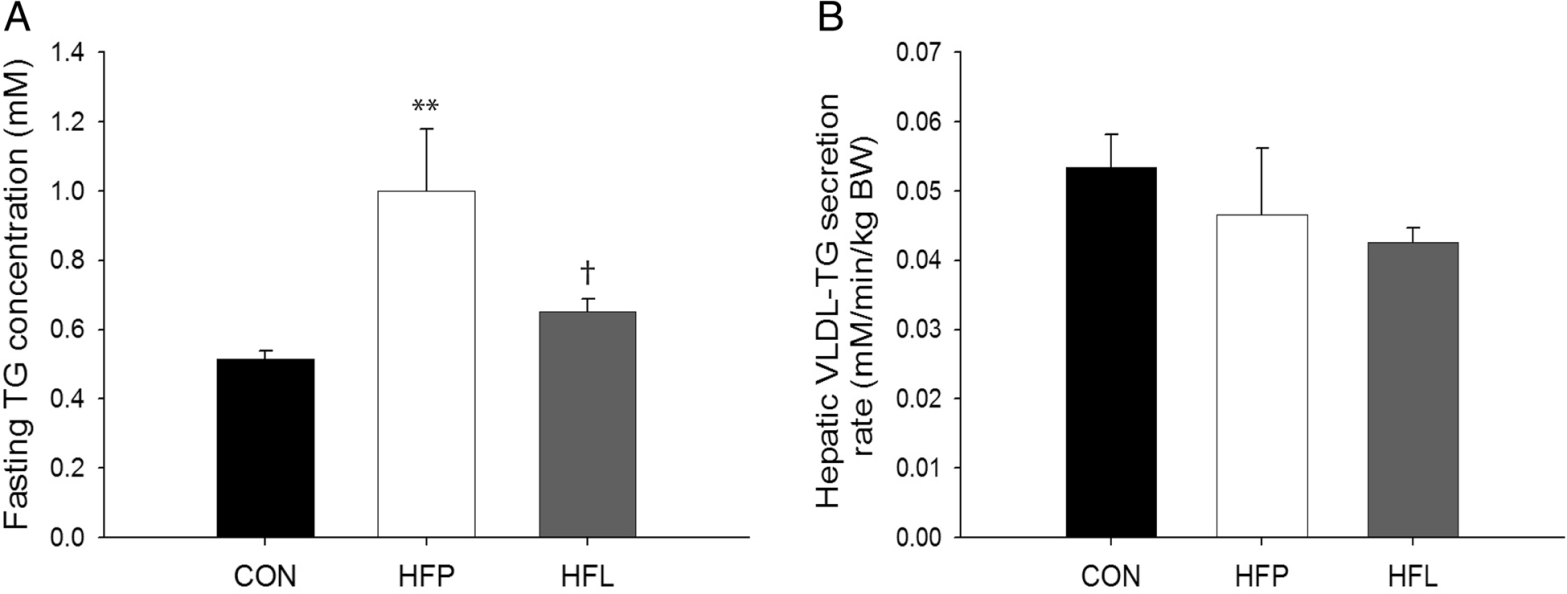
Hepatic very-low density lipoprotein-triglyceride secretion rate. Fasting plasma TG concentration (**a**) and rate of hepatic very-low density lipoprotein-triglyceride (VLDL-TG) secretion (**b**) in rats on normal chow control diet (CON), high-fat palm oil diet (HFP), and high-fat lard diet (HFL) (n = 11 per group). Data are expressed as mean ± SEM. ** *p* < 0.01 vs. CON, † *p* < 0.05 vs. HFP

Fasting plasma TG concentrations were higher in HFP animals compared with CON (*p* < 0.01) and HFL (*p* < 0.05) animals (Fig. 3a). After the administration of Triton WR1339, plasma TG concentrations increased linearly over time (mean R^2^ = 0.950 ± 0.008). Hepatic VLDL-TG secretion rates, as determined from the slope of the plasma TG concentrations versus time after administration of Triton WR1339, were however not affected by high-fat diet feeding (Fig. 3b).

### ipGTT with deuterated glucose

**Fig. 4:**
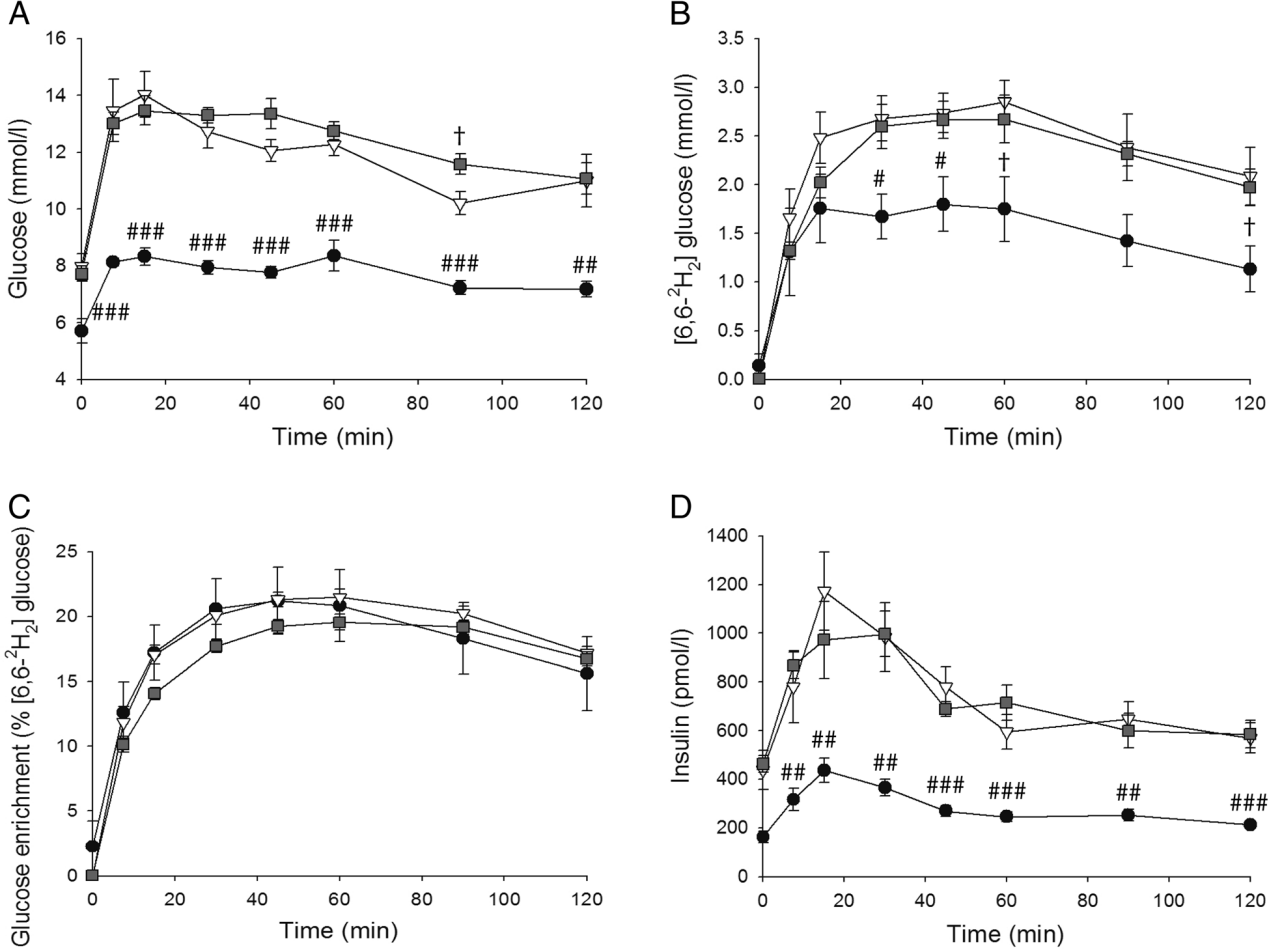
IpGTT with deuterated glucose. Time course of plasma glucose (n = 11 per group) (**a**), ^2^H_2_-glucose (n = 6 per group) (**b**), glucose enrichment (n = 6 per group) (**c**), and insulin (n = 11 per group) (**d**) during the ipGTT with deuterated glucose for rats on normal chow control diet (●), high-fat palm oil diet (∇), and high-fat lard diet (). Data are expressed as mean ± SEM. # *p* < 0.05, ## *p* < 0.01, ### *p* < 0.001 vs. HFP and HFL; † *p* < 0.05 vs. HFP

Data of the ipGTT with deuterated glucose are shown in Fig. 4 and the results are summarized in Table 3. Fasting plasma glucose was about 40 % higher in both high-fat diet groups compared with CON (*p* < 0.01; Table 3). The AUG_g_ was also higher in both HFP and HFL when compared with CON (*p* < 0.001) and, furthermore, AUG_g_ was higher in HFL than in HFP (*p* < 0.01). These results indicate the development of whole-body glucose intolerance in HFP and, to a larger extent, in HFL. Fasting plasma insulin was almost 3-fold higher in both high-fat diet groups compared with CON (*p* < 0.01) and the AUC_i_ was also higher in both HFP and HFL compared with CON (*p* < 0.01). Both high-fat diets led to the development of whole-body insulin resistance as indicated by the higher product of AUC_g_ and AUC_i_ (*p* < 0.01) and the higher HOMA-IR index (*p* < 0.01) in the HFP and HFL groups compared with CON. However, the measures for whole-body insulin resistance did not differ between HFP and HFL.

**Table 3:** Plasma glucose and insulin concentrations during ipGTT with deuterated glucose

	CON (n = 11)	HFP (n = 11)	HFL (n = 11)
Fasting glucose (mM)	5.7 ± 0.4	8.0 ± 0.5 **	7.7 ± 0.3 **
AUC_g_ (mM · h)	15.3 ± 0.5	22.5 ± 0.7 ***	25.6 ± 0.8 ***,††
Fasting insulin (pM)	163 ± 23	438 ± 82 **	463 ± 34 ***
AUC_i_ (pM · h)	670 ± 65	1622 ± 208 ***	1421 ± 112 **
AUC_g_*AUC_i_ (mM · h*pM · h)	9138 ± 689	41146 ± 6721 ***	36262 ± 2635 **
HOMA-IR	33.8 ± 4.8	134.8 ± 24.3 **	171.0 ± 13.3 ***
^2^H_2_-glucose concentrations (n = 6 per diet group)			
2 · AUC_d2g_ (mM · h)	6.0 ± 1.0	9.6 ± 0.9 *	9.1 ± 2.5
AUC_EN_ (% ^2^H_2_-glucose · h)	36.2 ± 4.9	37.5 ± 0.9	34.3 ± 1.0
Maximal EN (% ^2^H_2_-glucose)	22.6 ± 2.8	23.2 ± 0.7	20.9 ± 0.6
Time to maximal EN (min)	46.8 ± 4.2	52.8 ± 2.2	58.4 ± 1.6 *

Data are expressed as mean ± SEM. CON, normal chow control diet; HFP, high-fat palm oil diet; HFL, high-fat lard diet; AUC_g_, area under the glucose curve; AUC_i_, area under the insulin curve; HOMA-IR, homeostatic model assessment for insulin resistance; AUC_d2g_, area under the ^2^H_2_-glucose curve (a factor 2 is necessary for comparison of area under the ^2^H_2_-glucose curve to area under the glucose curve because the glucose injected was only 50 % enriched in ^2^H_2_-glucose); EN, glucose enrichment (% ^2^H_2_-glucose of total glucose); AUC_EN_, area under the EN curve. * *p* < 0.05, ** *p* < 0.01, *** *p* < 0.001 vs. CON; †† *p* < 0.01 vs. HFP

To determine the glucose tolerance of the liver, ^2^H_2_-glucose was used in the ipGTT. The AUC of plasma ^2^H_2_-glucose was higher in HFP compared with CON (*p* < 0.05), while it only tended to be higher in HFL compared with CON (p = 0.066; Table 3). The AUC of the glucose enrichment (EN) and the maximal EN did not differ between groups. However, the time to reach the maximal EN was longer in HFL than in CON (*p* < 0.05). Although the difference is small, this shows that in HFL animals the liver is producing more (unlabeled) glucose despite increased plasma glucose concentrations, indicating that the liver of HFL animals is slightly more glucose intolerant.

## Discussion

The present study aimed to elucidate the effects of low-stearate palm oil and high-stearate lard high-fat diets on liver lipid metabolism and glucose tolerance. To this end, Wistar rats were fed with a normal chow control diet (CON), a high-fat diet based on palm oil (HFP), or a high-fat diet based on lard (HFL) for a period of 10 weeks. Both high-fat diets resulted in an elevated IHCL content compared with CON, which was accompanied by a delayed uptake and/or slower turnover of dietary fat in the liver, but without any change in VLDL-TG secretion rates. Surprisingly, IHCL content was higher in HFP than in HFL, despite the increased fatty acid oxidative capacity in isolated liver mitochondria of HFP animals. In contrast, while both high-fat diets induced whole-body glucose intolerance, only HFL impaired hepatic glucose tolerance.

Ten weeks of HFP and HFL diet feeding in rats resulted in similar increases in energy intake and body weight gain compared with CON. In a previous study in mice using exactly the same diets for a period of 5 weeks, HFL led to a larger gain in body weight compared with HFP, which was accompanied by a lower energy expenditure, mainly due to a significantly lower fatty acid oxidation rate [25]. However, in another study 19 weeks of feeding high-fat diets with palm oil and lard oil in mice resulted in similar body weight gains [42]. Besides the differential effects of diets in different species and strains, also other factors such as starting age and duration of the diet, stress, and housing conditions are known to modulate the effects on body weight [43], which may explain the different results in the various studies.

Because stearate is inefficiently oxidized by hepatocytes [18–21] and because it is a poor substrate for esterification into TG and subsequent VLDL synthesis and secretion [22, 23], we hypothesized that HFL would induce greater hepatic steatosis as compared with HFP. In contrast, we found that, while both high-fat diets increased IHCL content compared with CON, the increase in IHCL was larger with HFP than with HFL. Moreover, we observed no differences in VLDL-TG secretion rates among the diet groups. We did however find that HFP feeding upregulated the capacity for fatty acid β-oxidation in liver mitochondria, as evidenced by the increased state 3 oxygen consumption rates with the fat-derived substrates palmitoyl-L-carnitine and palmitoyl-CoA, but not with the glucose-derived substrate pyruvate. In contrast, there was no significant effect of HFL feeding on liver mitochondrial capacity. Interestingly, at the whole-body level, HFP feeding has been shown to lead to higher fatty acid oxidation rates as compared with HFL feeding [25], which corresponds with the greater effects on liver fatty acid oxidative capacity in HFP animals in the current study. The increased capacity for fatty acid oxidation upon high-fat diet feeding has been regarded as an adaptive response to prevent the damaging effects of an increased influx of fatty acids into the liver [44–46]. However, apparently this adaptation in HFP animals was insufficient to cope with the dietary lipid overload.

Therefore, we also looked into the time course of uptake and utilization of dietary lipids in the liver, using in vivo ^1^H-[^13^C] MRS after the oral administration of a ^13^C-labeled lipid mixture. In CON rats, we observed a 4-fold increase in the ^13^C enrichment of the IHCL pool 4 h after ^13^C-labeled lipid administration (not shown), which is similar to our earlier results in healthy rats [30] and which is a measure for the uptake of dietary lipids by the liver. Thereafter, between 4 and 24 h after ^13^C-labeled lipid administration, ^13^C-labeled IHCL content decreased 2.5-fold in CON rats, indicating the turnover of diet-derived lipids in the liver through oxidation and secretion [30]. In comparison with CON, the dynamics of dietary lipid uptake and turnover in the liver was markedly different in the high-fat diet groups. In the early postprandial period, 4 h after the administration of the ^13^C-labeled lipids, dietary lipid uptake was lower in both high-fat diet groups compared with CON. However, between 4 and 24 h the ^13^C-labeled IHCL content did not decrease, but further increased, suggesting a delayed uptake and/or a slower turnover of dietary fat in the liver after high-fat diet feeding, which may have contributed to the development of liver steatosis. These results are in contrast with our findings in other rat models of liver steatosis, i.e. pre-diabetic and diabetic obese Zucker diabetic fatty rats, in which liver steatosis was associated with increased dietary lipid uptake in the early postprandial period, while the turnover of diet-derived lipids was largely unaffected [31]. The lower uptake of ^13^C-labeled lipids in high-fat diet fed rats in the early postprandial period could possibly be due to the higher dilution of the ^13^C label with the relatively high amount of unlabeled lipids from the high-fat diets (45 En% from fat) compared with the diets for CON rats (9 En% from fat) and Zucker diabetic fatty rats (19 En% from fat). However, in other studies it has been shown that both in type 2 diabetes patients and in streptozotocin-induced diabetic rats there is a delay in the removal of chylomicron remnants from the circulation [47–50], which is in agreement with our data of delayed dietary lipid uptake in the liver of high-fat diet fed animals. The molecular basis for the defective clearance of chylomicron remnants in diabetic conditions has not been elucidated, but it may be induced by insulin resistance and a consequent down-regulation of the low density lipoprotein (LDL) receptor, the latter of which is the primary route for chylomicron remnant removal by the liver [49, 51]. However, in streptozotocin-induced diabetic rats the delayed remnant removal seems to be associated with insulin deficiency rather than insulin resistance, as similar effects were observed with regular and high-fat diet feeding [50].

We found no major differences in the dynamics of dietary lipid uptake and turnover in the liver between the two high-fat diet groups. It should be noted that all animals received the same ^13^C-labeled lipid mixture, which does not match the composition of the different diets, and that the ^1^H-[^13^C] MRS method does not differentiate between different fatty acids. Therefore, these experiments do not provide specific information on the liver uptake of palmitate versus stearate, which is a limitation of the study. Stearate is poorly absorbed from the gut [52]; however, intestinal absorbability of stearate is similar to that of palmitate [53] and therefore the greater IHCL accumulation with HFP compared with HFL is not likely caused by differences in absorption. The increase in ^13^C-labeled IHCL content between 4 and 24 h after ^13^C-labeled lipid administration tended to be higher in HFP than in HFL (p = 0.07; Fig. 2d). Therefore the increased IHCL accumulation in HFP as compared with HFL may be associated with an increased uptake of dietary fat during the late postprandial period. Moreover, fasting plasma TG levels were higher in HFP than in HFL, which may have contributed to the higher IHCL accumulation in HFP through a mass action effect. Finally, increased *de novo* lipogenesis could also be a mechanism for the greater IHCL accumulation in HFP as compared with HFL, but to the best of our knowledge, there is no literature data on differential effects of low-stearate versus high-stearate diets on *de novo* lipogenesis in the liver.

Both high-fat diets induced hyperglycemia, hyperinsulinemia, whole-body glucose intolerance, and whole-body insulin resistance, but the impairment of whole-body glucose tolerance (determined from the AUC_g_ during the ipGTT) was slightly greater in HFL than in HFP. Moreover, the deuterated glucose tolerance test showed that in HFL animals the liver produces more (unlabeled) glucose despite increased plasma glucose concentrations, which indicates that in HFL animals the liver is more glucose intolerant than in CON and HFP animals. This is in agreement with the study of van den Berg et al., in which mice receiving a HFL diet developed both hepatic and peripheral insulin resistance, while mice fed with a HFP diet only developed peripheral insulin resistance [25]. Our findings question the role of IHCL in the development of liver insulin resistance [26], as HFP feeding resulted in a greater accumulation of IHCL than with HFL feeding, but without any signs of liver glucose intolerance. However, the greater IHCL accretion in HFP was accompanied by an increased mitochondrial capacity for fatty acid oxidation in the liver, which may have prevented the accumulation of toxic, lipid-derived intermediates, such as diacylglycerols, explaining the absence of lipid-induced glucose intolerance in the liver of HFP animals [26]. Alternatively, the slight impairment of hepatic glucose tolerance with HFL may be explained by specific effects of stearate on pathways intersecting with the insulin signaling pathway [54–57] or by its effects on membrane rigidity and insulin signal transduction [58–60].

## Conclusion

In conclusion, both high-fat diets based on palm oil and lard induced liver lipid accumulation, which was accompanied by a delayed uptake and/or slower turnover of dietary fat in the liver without any effects on VLDL secretion. However, despite the even larger accretion of lipids in the liver with the high-fat palm oil diet, only the high-fat lard diet impaired hepatic glucose tolerance.

## Abbreviations

AUC: area under the curve
CAT: carboxyatractyloside
CPT1: carnitine palmitoyltransferase 1
EN: enrichment
En%: energy percent
HOMA-IR: homeostatic model assessment for insulin resistance
IHCL: intrahepatocellular lipids
ipGTT: intraperitoneal glucose tolerance test
LASER: localization by adiabatic selective refocusing
LDL: low density lipoprotein
MRS: magnetic resonance spectroscopy
NAFLD: non-alcoholic fatty liver disease
NASH: non-alcoholic steatohepatitis
NEFA: non-esterified fatty acids
POCE: proton-observed, carbon-edited
RCR: respiratory control ratio
SWAMP: sequence for water suppression with adiabatic modulated pulses
TG: triglycerides
VLDL: very-low density lipoprotein
